# Analytical Review of Event-Based Camera Depth Estimation Methods and Systems

**DOI:** 10.3390/s22031201

**Published:** 2022-02-05

**Authors:** Justas Furmonas, John Liobe, Vaidotas Barzdenas

**Affiliations:** Department of Computer Science and Communications Technologies, Vilnius Gediminas Technical University, 03227 Vilnius, Lithuania; justas.furmonas@stud.vilniustech.lt

**Keywords:** event-based camera, neuromorphic, depth estimation, monocular

## Abstract

Event-based cameras have increasingly become more commonplace in the commercial space as the performance of these cameras has also continued to increase to the degree where they can exponentially outperform their frame-based counterparts in many applications. However, instantiations of event-based cameras for depth estimation are sparse. After a short introduction detailing the salient differences and features of an event-based camera compared to that of a traditional, frame-based one, this work summarizes the published event-based methods and systems known to date. An analytical review of these methods and systems is performed, justifying the conclusions drawn. This work is concluded with insights and recommendations for further development in the field of event-based camera depth estimation.

## 1. Introduction

Computer vision has been one of the most popular research areas for many years. Numerous applications exist where computer vision plays an important role, e.g., machine inspection, photogrammetry, medical imaging, automotive safety, etc. [1]. These applications each incur disparate problems though common methods have been utilized to solve these problems. For most machine vision applications neural networks have been employed, and through the years different frameworks have been created to help solve various problems faster and more accurately. In addition, numerous databases have been made available online that can train any neural network to solve most machine vision problems precisely without any additional training. Thus, computer vision has grown to a mature level and has been applied in a broad spectrum of fields.

On the other hand, computer vision to this day has extensively utilized frame-based cameras which have existed for many more years than computer vision itself. A frame-based camera outputs data corresponding to the captured light intensity at every selected pixel synchronously. This technology has been effective, and for many years has proven to be superior to any other camera type. Nevertheless, for many applications, frame-based cameras have features that impact performance and accuracy. Frame-based cameras suffer from high latency, low dynamic range, and in some cases high power consumption. For example, when using a frame-based camera to capture high-speed motion, the captured images will exhibit motion blur which would make image processing impossible or at the very least degrade the processing accuracy. Some solutions exist that can remove or mitigate motion blur or substitute it with motion flow using deep neural networks [2]. From a hardware perspective, another way to mitigate motion blur would be to increase the frame speed of a camera. However, this is not a trivial task. Besides the increased camera power consumption associated with operating at a higher frame rate, the data handling requirements of the associated image processor or digital signal processor (DSP) increase exponentially as well. Hence, frame-based cameras for many computer vision applications have significant challenges which have been difficult to overcome.

A potential solution to the aforementioned obstacles may come from the fast-growing field of neuromorphic computing. Neuromorphic computing consists of a variety of brain-inspired computers, devices, and models that contrast the pervasive von Neumann computer architecture [3]. This field inspired researchers in search of a more biological approach to computer vision, one of which is a silicon retina. A neuromorphic image sensor works asynchronously and typically extracts spatiotemporal information from the scene much like other biological vision systems. The objective of these sensors is to use techniques employed by biology to improve data acquisition, sensitivity, dynamic range, and spatial and temporal resolution [4]. Because of this non-standard principle of operation these neuromorphic image sensors output sparse event data. Most of the methods and algorithms that have been developed for frame-based computer vision are no longer suitable in event-based vision systems. Moreover, the large datasets available for training and characterizing frame-based vision systems are not usable for the event-based variety due to the differing data output.

A new type of computer vision is on the rise and may be the candidate to outperform frame-based vision for many applications. In this paper, the main characteristics of an event-based camera, its data output, and the methods that are used to process this data are introduced and explained. For this work, “event-based”, “neuromorphic”, and “dynamic” will be used interchangeably to describe the same bio-inspired camera. Once the reader understands the basics of an event-based camera, an overview of currently published methods for stereo and monocular depth estimation by event-based cameras from a hardware perspective is discussed. In summary, the depth estimation results of these different methods are compared, and conclusions are formulated.

## 2. Event-Based Cameras

In this section, a brief introduction of the event-based camera itself with a summary table of the state-of-the-art sensors is presented. The main properties of the sensor, a brief overview of the pixel hardware, and the output of the sensor are discussed. Lastly, general applications of an event-based camera are presented.

The event-based vision system is a novel type of camera with, most notably, an asynchronous data output. Like the conventional camera, the event-based vision or image sensor is composed of pixels, but whereas typical image sensors capture intensity, event-based image sensors detect intensity changes (both positive and negative) in the scene above some user-defined threshold. Thus, event-based image sensors capture “events” as they occur without any timing synchronization. Moreover, these events are captured without the necessity of also capturing intensity values for the utilization of frame differencing techniques as is typically performed with conventional frame-based cameras. This pixel-level asynchronous, event detection is the main breakthrough that allowed event-based cameras to gain traction outside of the academic community.

There have been many attempts to create the optimal pixel and accompanying readout structure for the realization of an event-based camera. Not many of the early event-based sensors succeeded because of the exceedingly high noise floor. Most of these preliminary offerings exhibited high noise levels that rendered them useless for commercial deployment. The most successful event-based image sensors have reached the commercial market and are readily available to researchers. Currently, there are five major players for event-based vision sensors: Prophesee, iniVation, Samsung, CelePixel and Insightness. Table 1 summarizes some features of the event camera offerings from these vendors.

The major differentiating performance parameters of event-based vision sensors are latency, dynamic range, power consumption. The latency between triggered events has dropped to less than 0.5 μs [5,6]. The non-linear dynamic range is fairly the same in all the presented research papers with a maximum value up to 143 dB [7]. Power consumption is measured with 100 kevents/s and drops to 0.25 mW at a supply voltage of 1.2 V [8]. Another important measure is the bandwidth which was reported at the theoretical maxima of 1.3 Gevents/s [9]. The biggest drawback of these sensors is the pixel size. The pixel pitch needs to be decreased to achieve yields like that of standard CMOS image sensors [10]. Currently, the smallest pixel pitch of a commercially available event-based sensor is 4.86 μm^2^ [11]. As seen in Table 1, only recently has the focus turned to pixel size reduction.

**Figure 1:**
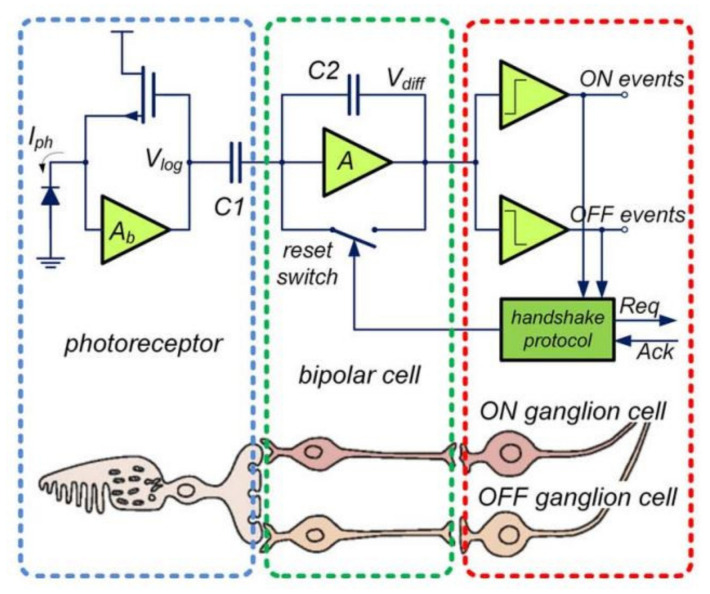
Three-layer model of a human retina and corresponding DVS pixel circuitry (left) [13].

The main design principle of an event-based pixel is to mimic the performance of the mammalian retina. The mammalian retina is a complex structure that is composed of ten layers in total [12]. The structure and density of light-sensitive cells, bipolar neurons, ganglion cells, and others are too complex to replicate in silicon, hence the pixel realization requires simplification. The simplified, event-based pixel is composed of three layers: the photoreceptor layer, the outer plexiform layer, and the inner plexiform layer [13]. These three layers have a complexity of cells which are further reduced to the cone or rod photoreceptors, ON/OFF bipolar cells, and ON/OFF ganglion cells. The combination of these cells is the optimal structure for a silicon retina that can be utilized for most imaging applications while still mimicking the mammalian retina. The silicon equivalent replicates this structure by introducing a photoreceptor, a differencing circuit (bipolar cells) and a couple of comparators (ganglion cells) [14]. The circuit realization is shown in Figure 1. This structure was also adopted to create the first very-large-scale integration (VLSI) silicon retina [15]. Since then, the silicon retina pixel architecture has become standard and has not been changed very much, like the active pixel sensor (APS) pixel architecture that is utilized in standard CMOS image sensors [16].

**Table 1:** Summary of published event-based vision systems.

Reference	Resolution	Latency (μs)	Dynamic Range (dB)	Pixel Size (μm^2^)	Power Consumption (mW)	Supply Voltage (V)
[15]	128 × 128	15	120	40 × 40	24	3.3
[7]	304 × 240	3	143	30 × 30	175	3.3
[17]	128 × 128	3.6	>100	35 × 35	132–231	3.3
[18]	64 × 64	-	-	33 × 33	15	3.3
[19]	128 × 128	3	120	30 × 31	4	3.3
[20]	240 × 180	12	120	18.5 × 18.5	7.4–13.5	3.3
[21]	240 × 180	3	130	18.5 × 18.5	5	3.3
[22]	640 × 480	<200	>80	9 × 9	27	2.8
[6]	768 × 640	<0.5	>120	18 × 18	-	-
[23]	346 × 260	-	-	18.5 × 18.5	-	-
[24]	320 × 262	1000	>100	13 × 13	70	-
[8]	132 × 1024	-	-	10 × 10	0.25	1.2
[5]	1280 × 800	<0.5	>120	9.8 × 9.8	400	3.3
[11]	1280 × 720	<200	>124	4.86 × 4.86	32	2.8
[9]	1280 × 960	-	-	4.95 × 4.95	150	2.8
[24]	800 × 600	1000	>100	7.2 × 7.2	250	-

### 2.1. Event-Based Camera Data Coding

The spike (event) encodes information of light intensity changes in a scene. A scene’s reflectance change generates the needed voltage output from a photoreceptor that passes a configurable threshold, and an event (spike) is generated. The comparators (ganglion cells) are responsible for discerning whether the detected event surpasses the configurable threshold, emitting ON and OFF events for readout. Thus, if an ON event is generated, then the intensity has increased, and if an OFF event is generated, then the intensity has decreased.

On its own, the spiking information is not enough for a digital representation due to missing information of when and where the event was generated. Hence, the address-event representation (AER) protocol is used to encode this additional information in a lightweight form that is easy to use by other processes. Every pixel has an *x*, *y* address which usually is encoded with enough bits for the resolution of the event camera. The polarity of a pixel event (1 or 0) is encoded with 1 bit. In addition, a timestamp of an event is also added, which is by default 32 bits for most realizations. Thus, an AER protocol is used as an output of a dynamic vision sensor (DVS). The event-based sensors generate a stream of events that can be expressed as:

*e*_i_ = {*x*_i_, *y*_i_, *p*_i_, *t*_i_}, (1)

where *x*_i_, and *y*_i_ are the coordinates of an active pixel, *p*_i_ is the polarity of an event, *t*_i_ is the timestamp, and *e*_i_ is the *i*_th_ event in the stream.

The event does not provide a lot of information, but a group of events (event stream or train) form a spatiotemporal shape of the object. Here, the spatiotemporal resolution is defined by spatial resolution, which usually is determined by hardware limitations, i.e., the number of pixels, and the temporal resolution is defined by the speed of event acquisition. Event cameras show a microsecond latency which incorporates a good temporal resolution. This temporal resolution was already adopted by the HFirst algorithm which is used for object recognition [25]. Later, time surfaces (hierarchy of time surfaces (HOTS) and histograms of averaged time surfaces (HATS)) were presented, which used the spatiotemporal resolution provided by the event-based cameras to generate 3D maps of event data [26,27]. However, object recognition applications are not the only area that may benefit from the temporal resolution of the event camera. Depth from defocus (DFD) has been presented, which uses the temporal resolution of the event-based sensor in combination with spiking neural networks (SNN) to calculate the depth of a scene [28]. Thus, spatiotemporal features can be exploited in any visual task. Using the high temporal resolution of the event-based image sensor can bring new research in the artificial vision field.

### 2.2. Events and Spiking Neural Networks

The nature of event-based cameras and their high temporal resolution correlates well with spiking neural networks (SNNs). SNNs work in a very similar way to biological neural networks. Moreover, SNNs behave differently than the well-known standard artificial neural networks (ANNs). ANN-neuron computations are performed in a synchronous manner:

(2) $$y(n)=f\;(\sum_{i=1}^{k}x_{i}\left(n\right)w_{i}\left(n\right)+b_{i}\left(n\right)),$$

where *y* is the output of the neuron, *f* is the activation function, *x*_i_ is the *i*_th_ input of the neuron model, *w*_i_ is the *i*_th_ weight, *k* the number of neurons, *n* the discrete-time moment, and *b*_i_ is the *i*_th_ bias.

**Figure 2:**
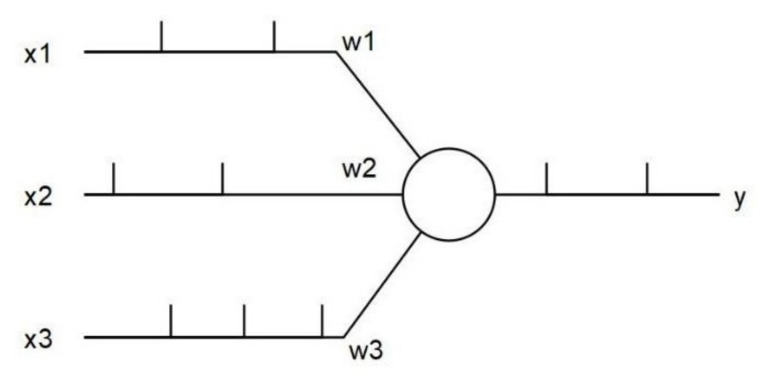
A simplified SNN neuron model.

The SNN model depends on the asynchronously generated event stream. Figure 2 shows a simplified SNN neuron model where *x*_1_, *x*_2_ and *x*_3_ are inputs of the neuron; *w*_1_, *w*_2_ and *w*_3_ are weights; and *y* is the output of the neuron. The spikes are seen on the input and output. The action potentials are travelling on the input lines at different timestamps. The neuron accepts the inputs and generates an output in an asynchronous manner.

The main differences between SNNs and ANNs are well summarized in [29]:

1. The ways in which information is encoded in SNNs and ANNs are different. A non-spiking neuron uses real-value activations to convey information whereas a spiking neuron modulates information on spikes.
2. A non-spiking neuron in an ANN does not have any memory, yet spiking neurons typically have memory.
3. The output generated by many ANNs, especially feedforward ANNs, is not a function of time, yet most SNNs are time-varying in nature.

These three statements confirm how event-based cameras and their associated high temporal resolution correlates well with SNNs. The events of a dynamic vision sensor can be collected into a 3D structure that captures spatiotemporal information. Moreover, this information cannot be redundant because pixels detect only temporal changes, thus resulting in a sparse representation of the scene. At every pixel location, illuminance log intensity changes generate asynchronous events which arrive at sub-microsecond accuracy in the form of AER data. This temporal accuracy provides precise spike timing information, which can be exploited by an SNN even though the output of the vision sensor is digital (in the form of AER data) [25]. A spiking neuron model is used to generate an output spike which would subsequently be fed into an SNN.

There is a variety of spiking neuron models. The first model presented here is not used in practice due to its inefficiency. The other two models are used more prevalently. The important aspect of using a neuron model is to generate spikes. These spikes are then fed into a spiking neural network. A spike is an active membrane potential, and it is generated when a certain membrane voltage threshold is crossed. After the neuron fires, it is inactive for the refractory period.

#### 2.2.1. Hodgkin–Huxley Model

This is one of the first models that describes the behaviour of biological neurons [30]. The Hodgkin–Huxley spiking neuron model is a building block of understanding how current flows in the neuron, but this model is too complicated to be implemented in silicon. The total membrane current *I* can be represented as a function of time and voltage:

(3) $$I=C_{M}\frac{dV}{dt}+g_{K}n^{4}(V-V_{K})+g_{\mathrm{Na}}m^{3}h(V-V_{\mathrm{Na}})+g_{l}(V-V_{l}),$$

where *C*_M_ is membrane capacity per unit area, *V* is membrane potential displaced from the resting potential, and *t* is time. The terms *V*_Na_, *V*_K_ and *V*_l_ are called reverse potentials and can be measured directly as displacements from the resting potential, *g*_K_, *g*_Na_ and *g*_l_ are the conductance of potassium, sodium, and the leakage channel, respectively, and *n*, *m* and *h* are dimensionless quantities whose variations with time after a change of membrane potential are determined by other equations.

The total membrane current is made of four components on the right-hand side: the capacity current, the current carried by K ions, the current carried by Na ions and the leakage current, which is made up of chloride and other ions. Here, ionic permeability is expressed in terms of ionic conductance. The ionic conductance of each ionic current component is elaborated in greater detail in the same paper. Thus, a conclusion can be drawn that current can be carried through the membrane either by charging the membrane capacity or by the movement of ions in parallel with the capacity.

#### 2.2.2. Leaky Integrate-And-Fire Model

This is a simpler model compared to the Hodgkin–Huxley model, hence it can be implemented in VLSI. The Leaky Integrate-And-Fire (LIF) model is one of the most popular spiking neuron models used to simulate SNNs. The LIF model can be mathematically written as follows:

(4) $$\tau_{m}\frac{dv_{m}\left(t\right)}{dt}=-(v_{m}(t)-E_{r})+R_{m}I(t),$$

where *τ*_m_ is the membrane time constant, *v*_m_(*t*) the membrane potential, *E*_r_ is membrane resting potential (const.), *R*_m_ is membrane resistance, and *I*(*t*) is the sum of the current supplied by the input synapses. Equation (4) indicates that when *I*(*t*) = 0 the membrane potential exponentially resets to the resting potential, *v*_m_(*t*) = *E*_r_, with time constant *τ*_m_. Here, the exponential decay is due to membrane capacitance charge over time. This time is also important after a spike is generated and is usually called the refractory period.

When the membrane voltage *v*_m_(*t*) reaches the threshold level *V*_th_ an output spike is generated, the membrane voltage drops lower than *E*_r_ and after the refractory period resets to *E*_r_. The current supplied by the input synapses can be expressed as:

*I*(*t*) = *W* · *S*(*t*), (5)

where *W* = [*w*_1_, *w*_2_, …, *w*_N_] is the weight vector and *S*(*t*) = [*s*_1_(*t*), *s*_2_(*t*), …, *s*_N_(*t*)] is the spatiotemporal input spike pattern containing *N* input spike trains. Hence, the weights are used together with input spikes to produce an input current strong enough to trigger a neuron to output a spike. This will ensure that only the strongest connections can trigger a neuron to fire. These spike trains are heavily dependent on time and can be expressed as:

(6) $$S_{i}(t)=\sum_{f}\delta \left(t-t_{i}^{f}\right),$$

where $t_{i}^{f}$ is the firing time of *i*_th_ neuron, *f*_th_ is a spike in the input spike train, and *δ* is the Dirac function. As seen from this equation the input spikes are reduced to points in time. Thus, the LIF model is most useful in many artificial SNNs due to its simplicity.

#### 2.2.3. Izhikevich Model

For some applications, the LIF model does mimic biological neurons very accurately. An intermediate complexity choice is the Izhikevich model [31]. This spike model has a two-dimensional (2D) system of ordinary differential equations of the form:

(7) $$\frac{dv}{dt}=0.04v^{2}+5v+140-u+I,$$

(8) $$\frac{du}{dt}=a(bv-u),$$

with the auxiliary after-spike resetting, i.e., if *v* ≥ 30 mW, *v* is reset to *c* and *u* is reset to *u* + *d*. Here, *u,* and *v* are dimensionless variables, *a*, *b*, *c*, and *d* are dimensionless parameters, and *t* is the time. The variable *v* represents the membrane potential of the neuron and *u* represents the membrane recovery variable.

These equations were designed to simplify the Hodgkin–Huxley neural model as well as produce different firing patterns similar to those found in real biological neurons. Part of Equation (7) was obtained by fitting the spike initiation dynamics so that the membrane potential *v* would have a *mV* scale, and the time *t* would have *ms* scale. The variable *a* controls the recovery time. Parameter *b* controls the sensitivity of the recovery variable *u*, which could lead to subthreshold oscillations and low threshold spiking dynamics. The parameter *c* describes the after-spike membrane potential. Finally, parameter *d* describes the after-spike reset of the recovery variable *u*. This model is the simplest of the existing spiking neuron models consisting of only two equations and one nonlinear term.

In conclusion, the AER protocol output of asynchronous events can be used in combination with SNNs. The generated events stimulate the membrane voltage to pass the threshold and generate a spike. As we will describe in later sections, SNNs are already being implemented with event-based camera data and are demonstrating promising results. Additionally, these implementations are highly superior in terms of latency and power consumption compared to ANN-based implementations.

### 2.3. Event-Based Camera Characteristics

Bio-inspired vision sensors have many advantages as compared to traditional frame-based cameras. These advantages are a direct result of their hardware design. The main performance characteristics of event-based cameras are listed below:

- Power consumption: The dynamic vision sensor outputs only event data when objects in the scene move with respect to the camera or the camera itself moves. The events are generated at the edges of objects, i.e., where the light intensity changes. This characteristic drastically reduces redundant data. When the scene is static and objects in the scene do not move, no output is generated, except for the noise from individual pixels. However, this noise can be filtered out using background filtering techniques [32].
- Latency: The latency of the dynamic vision sensor is closely correlated with the asynchronous behaviour of the pixel. Conventional cameras need to synchronously capture every pixel’s data and then pass this data to some processing unit. The DVS event is asynchronous, and the subsequent processing unit time step is on the order of microseconds, as limited by the analogue circuitry comprising the pixel, not the integration time. Hence, it can be extensively used in safety-critical applications like those related to the automotive industry [33].
- Dynamic range: In event-based cameras, the dynamic range is 120 dB (or even more) as compared to frame-based cameras— 60 dB. In the biological retina, the adaptation to light level already starts in photoreceptors. It eliminates the dependency on absolute lighting level and instead the receptors respond to changes in the incident light (also known as temporal contrast) [13,34]. In silicon, this is based on a compressive logarithmic transformation in the photoreceptor circuit [14].

These three performance parameters are the main characteristics that differentiate event-based cameras from conventional frame-based ones. However, the neuromorphic vision system still lacks processing algorithms that offer similar capability as demonstrated with conventional frame-based algorithms. One of the application spaces where these major advantages can be better leveraged is depth-sensing. The following sections present depth-sensing algorithms that were created using different hardware configurations.

## 3. Event-Based Depth Estimation in Hardware

Depth estimation in computer vision is not a trivial task. There are two predominant techniques of getting the depth information from a scene— static or active. Active depth estimation uses an active source to measure depth, like a laser in light detection and ranging (LIDAR) or laser detection and ranging (LADAR) systems, and requires a sensor receiver. Active depth estimation techniques are computationally heavy, as well as expensive and not convenient for size, weight, and power-constrained environments. Static depth estimation does not need an active source. A stereo vision rig with multiple cameras can be used, which solves the correspondence problem and outputs a disparity map. On the other hand, stereo ranging might be too complex for an embedded robotic system with limited embedded computational resources. Embedded systems require modern solutions exhibiting low power consumptions, low data rates, and minimum latencies. Monocular depth estimation with an event-based camera might be a viable solution.

While event-based cameras have more advanced and efficient characteristics compared with their frame-based counterparts, it is still important to have an optimized computational platform. Firstly, the utilized computing device must be able to capture data coming off an event-based camera with low latency. Moreover, this data must be processed efficiently to extract accurate depth information from a scene in a timely manner. In addition, the design process with the selected hardware should be flexible and scalable enough for future improvements as application requirements perpetually increase. However, the processor should also be a low-power device so that the event-based camera’s power consumption can have a meaningful impact on the aggregate system. Therefore, the performance parameters of the utilized hardware play an important role in the performance of the amassed depth-estimation system. We further review depth estimation methods based on the hardware that was used and summarize the review in two tables. In Section 3.1 we present methods carried out with field-programmable gate arrays (FPGAs), in Section 3.2 we introduce methods developed with a neuromorphic platform, and finally, in Section 3.3 we review methods implemented with a standard central processing unit (CPU).

### 3.1. Field-Programmable Gate Arrays

An FPGA is an all-around platform that can provide high efficiency and low power consumption simultaneously. An FPGA better balances flexibility and energy efficiency compared to a standard CPU or an application-specific integrated circuit (ASIC). More flexible solutions can be achieved with a CPU and better energy efficiency can be realized using an ASIC. An FPGA can also be quite cheap compared with the ASIC fabrication and design process costs. The most important aspect of FPGAs for machine vision applications is the efficient real-time computation that FPGAs provide. Thus, FPGAs are pervasively utilized in many real-time machine vision deployments.

An FPGA paired with an event-based camera might be a commodity in the not-so-distant future. The event-based camera outputs asynchronous events coded in AER packets in digital format. This raw data can be sent from a sensor to an FPGA directly with no additional converters needed. This combination does not suffer from added latency and thus maintains the temporal resolution of the event-based camera at its minimum (smallest time-step). Although, to the best of the authors’ knowledge, just one paper presents a depth estimation solution implemented with an event-based camera and an FPGA. The method used a stereo vision rig and calculated disparities in microsecond-scale latency. In the following subsection, this method is briefly discussed.

#### Stereo Matching with Two Asynchronous Time-Based Image Sensors

This depth-sensing implementation uses an FPGA to handle the high temporal resolution data from both cameras positioned in stereo. During fast motion, massive streams of events need to be processed. Events are generated at sub-microsecond latencies and can reach densities of up to 1.3 Gevents/s. It should be noted that the data stream itself is already reduced because of the sparse nature of an event camera. These high data rates can be handled in real-time very readily with an FPGA. The stereo camera and FPGA configuration utilized by this paper reports distance measurement updates at 1140 fps [35].

**Figure 3:**
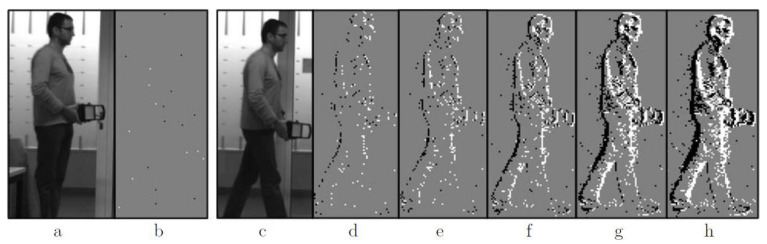
Silicon retina sensor in comparison to a conventional monochrome sensor. White pixels (off-events), Black pixels (on-events), Gray pixels (no events). (**a**) Person without movement in front of the monochrome sensor, (**b**) silicon retina output without movement, (**c**) person walking in front of the monochrome sensor, (**d**–**h**) silicon retina data from the walking person with collected events over a time period of 5 ms, 10 ms, 20 ms, 40 ms and 60 ms [35].

Two asynchronous time-based image sensors (ATIS) with a spatial resolution of 304 × 240 pixels, a temporal resolution down to 10 ns, and a dynamic range of 143 dB were used. Data from the camera was captured in AER packet format, which shows events’ *x* and *y* coordinates, polarities and timestamps. The FPGA handled the sparse and asynchronous data and computed disparity maps in real-time. However, the methodology proposed in this paper does not compute depth from individual pixels and requires the accumulation of events before performing any computations. This is a common practice when a time window is used to build up edges from individual events of an object. Additionally, a time period of events must be carefully selected to get complete object contours, but without blurred object edges. Therefore, the time period cannot be a fixed parameter, because it depends on the scene dynamics. Figure 3 shows the difference between multiple time periods.

This implementation, like many others, solved the correspondence problem to compute real-time depth maps. Here, a correspondence search is not performed on each pixel individually, but for a pixel block with a predefined size—16 × 16 pixels. Moreover, because the pixels work asynchronously, a correspondence search is performed only for image fields with a certain activity. This approach has two main benefits: (1) computational load is minimized; and (2) minimum memory accesses, resulting in a more efficient parallel memory architecture. Hence, the new event data from left and right sensors are stored in the memory as well as a filtered history of past events. It should be noted that the events having the same spatial location are overwritten when some predefined time threshold is passed. Later, segments of events are forwarded to a matching pipeline, followed by a consistency check which outputs disparity maps.

In hardware, this algorithm was realized by dividing it into a read and write interface, a forward rectification unit, a prioritization and scheduling unit, a memory controller, a history managing unit, a matching pipeline, and a consistency check unit. After the rectification, incoming events are segmented by calculating the segment number from the pixel’s coordinate. For each segment, a counter is dedicated to managing the history of events; expired events are deleted. Segmentation (prioritization) is needed to calculate disparities. The matching pipeline is achieved in three steps; loading *L*, weighting *W*, and aggregation *A*. First, the left image segment with a corresponding right image disparity range is loaded. After that, events are weighted together using their timestamps. Finally, the maximum weight in the disparity range is determined. In the end, a consistency check is performed, and a resulting disparity map is outputted.

The algorithm was evaluated in two real-world indoor scenarios; a rotating disc and moving persons. The average distance error in depth, calculated from a comparison with ground truth data, was used. The rotating disk and two walking persons were at 1.5 m, 2.5 m, and 3.5 m, respectively, from the event-based cameras in a stereo configuration. Different correlation windows were also used (5 × 5, 9 × 9, and 15 × 15 pixels) in combination with varying history lengths (5, 10, and 15 ms for the first scenario and 20, 25, and 30 ms for the second scenario). The correlation windows did not have a significant impact in the first scenario, but for the second scenario, a larger correlation window resulted in a smaller depth error. Longer history lengths have also led to smaller depth errors. The average depth error reported for the rotating disk scenario was about 0.1 m and for the walking persons scenario was about 0.5 m.

Although the results regarding depth error are not so ground-breaking, the important aspect here was the performance of the algorithm using FPGA hardware. Execution time and data throughput mainly depend on the segment size, the size of the correlation window, and the disparity range. To calculate the disparity for one segment 1370 clock cycles were needed. This overhead was based on an image plane of 128 × 128 pixels, a segment size of 16 × 16 pixels, a window size of 5 × 5 pixels, and a disparity range of 36 pixels. If a 100 MHz clock is to be used, then the calculation time is only 13.7 μs. Even if all 64 segments must be calculated a frame rate of 1140 fps can be achieved. Thus, real-time depth estimation can be achieved using an FPGA.

In this section, the FPGA-based depth estimation method was briefly discussed. It showed high performance and low latency while computing disparity maps in real-time [35]. On the other hand, little research has been performed in depth-sensing using a combination of an event-based camera and an FPGA. One of the many reasons for this lack of research activity could be the associated complex and inflexible design process compared with that when using a standard CPU platform. Secondly, the application of spiking neural networks with event-based cameras has brought promising results from researchers. SNNs are inherently directly adaptable or well-combined with the new neuromorphic processors, whereas integration with FPGAs pose more rigorous integration challenges. Neuromorphic processors are capable of fast parallel processing and are low-power, which makes them perfect competitors for FPGAs. In the next section, we discuss the methods that were implemented and tested using neuromorphic hardware.

### 3.2. Neuromorphic Processors

SNNs have recently received attention as a novel, more efficient artificial neural networks that behave more like biological neurons. SNNs continue to replace traditional ANNs and exhibit superior performance as compared with traditional artificial methods. Moreover, neuromorphic computing has reached some significant milestones, which have been well summarized in [3]. At the time of publication for this paper, a few neuromorphic processors have already been manufactured and well-utilized: TrueNorth [36], Loihi [37], SpiNNaker [38], and some others still in development [39]. These neuromorphic processors are based on a non-Von Neumann computer architecture and can be well suited with an event-based camera, because of the spiking, asynchronous output of events. The following subsections summarize depth-sensing methods implemented with neuromorphic hardware processors.

#### Disparity Maps from Fully Event-Based Systems

The correspondence problem with a neuromorphic computing platform, SpiNNaker, was first solved by [40]. Although SpiNNaker is a neuromorphic computer based on a mesh network of low-power ARM processing cores, it still can be used as a simple neuromorphic processor. SpiNNaker is fully asynchronous and uses Leaky Integrate-And-Fire neurons. A cooperative network approach was extended into SpiNNaker’s spiking neural network. The system could reconstruct visual depth information at a fixed latency of 2 ms. However, no metric depth comparison with ground truth was reported. Power consumption, because of the SpiNNaker processor, reaches 90 W, rendering this system unusable in most real-world scenarios.

An event-based camera combined with TrueNorth has successfully demonstrated the ability of a fully event-based stereo system [41]. This approach was the first one (to the authors’ knowledge) to implement a fully event-based system. It used an event-based camera, and its output data was connected to nine TrueNorth chips. This implementation was able to calculate 400 disparity maps per second and even reached 2000 disparity maps per second with some trade-offs. This stereo system also has low power consumption (single passive/active chip 34.4 mW/35.8 mW (from a 0.8 V supply)) due to the disabling of neurons when the DVS does not capture any motion.

In [41], the program that is running on TrueNorth was written in the Corelet programming language that was specifically created by IBM for this hardware platform. This neuromorphic chip consists of systems of equations that define the behaviour of TrueNorth’s neurons. These equations are encased in modules called corelets. The corelets also have inputs and outputs that are interconnected to create an aggregate system. The study in [41] has seven major corelets that are all assigned a specific task.

**Figure 4:**
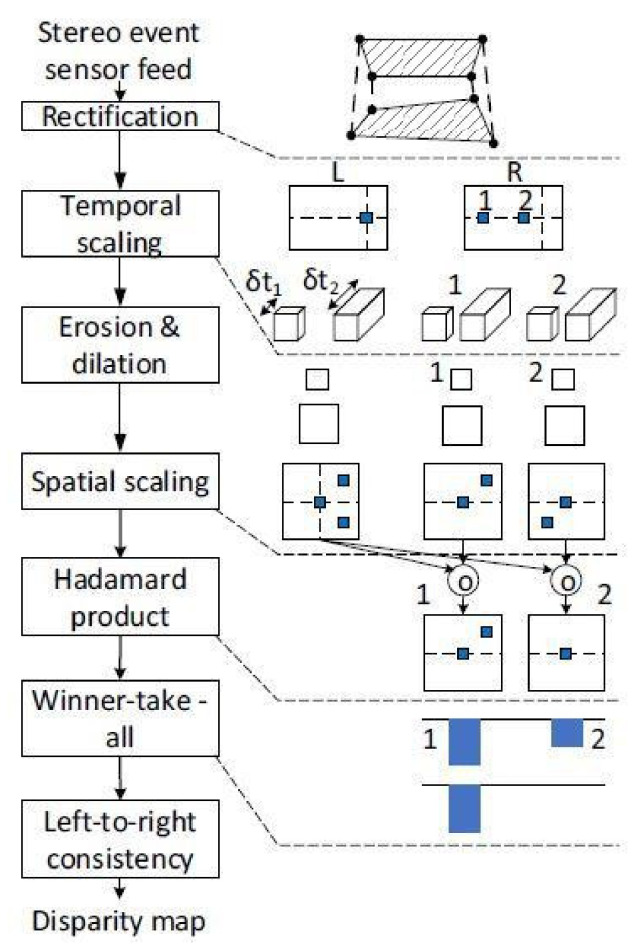
The pipeline of execution using input events generated by left and right event-based sensors [41].

First, the rectification corelet is used to map each pixel in the left and right sensor’s rectified space, to a pixel in the left and right sensor’s native resolution, respectively. Later, the multiscale temporal representation is used to add invariance across event rates because event rates are dynamic. Next, morphological erosion and dilation are used to denoise the image. The neuron potentials are initialized to zero and set to zero upon producing a spike. Later, spatial scaling is applied in order to extract coordinates where events form a feature vector. The Hadmard product is calculated for each pair of spatiotemporal coordinate tensors and is used for matching. Subsequently, the Hadmard product is converted to thermometer code. Finally, a winner-take-all feed-forward neural network is implemented which takes the generated thermometer code and finds the disparity with the largest value at every process clock tick. In the end, a left–right consistency check is performed to check whether the left-rectified pixel (p) matches a right-rectified pixel (q) and vice versa. The process is illustrated in Figure 4.

The algorithm was tested with a random-dot stereogram (RDS) dataset which includes a rotating synthetic 3D object, fast-rotating fan, and a rotating toy butterfly. The spike injection rate is up to 2000 Hz, while the disparity output is 400 Hz. The values for single-chip power consumption in passive and active mode are: 34.4 mW/ 35.8mW (0.8 V) and 82.1 mW/56.4 mW (1.0 V), respectively. Notably, the active mode in the latter case has smaller power consumption as compared to the passive mode case, with no explanation given by the authors. This may just be an organizational mistake as the lower power consumption in active mode does not intuitively match expectations. The neuromorphic processor shows reasonable results, outputting 400 disparity maps a second with a system latency of only 9 ms. The rotating fan and butterfly sequence had the smallest relative errors of 5–11.6% and 7.3–8%, respectively.

In conclusion, this section described depth-sensing methods that were developed and tested on neuromorphic processors. The first introduced method used a SpiNNaker processor, which runs on 90 W of power and thus would be challenging to use in most real-world applications. The second method utilizing a TrueNorth processor realized a fully event-based system that achieved high computational efficiency and low power consumption. Like FPGA-based implementations, little research has been performed in the area of depth estimation using neuromorphic hardware. Nevertheless, this should change soon as these processors become more widely accessible to the academic and research communities. The optimized integration of a neuromorphic processor with an event-based camera is expected to have a significant impact in the computer vision research field.

### 3.3. Standard Processors

A standard processing unit like a CPU or graphics processing unit (GPU) is commonly used to build flexible systems. However, they do not provide the best results, in terms of power consumption, latency, and computational efficiency. Still, CPUs and GPUs are the most popular choice among researchers when implementing event-based depth estimation methods. Interestingly, some of the methods developed on standard CPUs can be ported with minor corrections to a FPGA or even a neuromorphic processor. The following subsections are divided based on whether a stereo or monocular depth estimation method was utilized.

#### 3.3.1. Correspondence Problem in Stereo Depth-Estimation

Typically, the most popular methods for depth estimation utilize stereo vision. This is not surprising as solving the correspondence problem is well-developed using frame-based computer vision with many open-source algorithms readily available. One of the first to solve the correspondence problem with event-based vision sensors was [42]. The researchers in [42] used an embedded device with a DSP, which utilized a simple algorithm for stereo processing— camera calibration and rectification, stereo correspondence calculation and reconstruction (disparity map). Similar methods were later used, which evaluated the effect of the adjustment of the temporal resolution of the silicon retina on the correspondence calculation. The authors of [43] solved the correspondence problem using two time windows and implemented the algorithm with Java tools for AER (jAER) software. Their study [43] produced average errors up to 5.79% at its maximum tested distance of 60 cm. Going further, a spatiotemporal adaptive cooperative approach was used to calculate disparities for each incoming event in [44,45]. This approach showed a similar performance at short distances with a reported 95–98% accuracy.

The downside of the stereo vision system is the calibration process. A calibration error will impact the depth estimation results. Many stereo vision rigs focus on 3D perception/representation with some using 6 degrees of freedom (DOF) tracking [46,47,48,49,50]. The study in [51] presents two algorithms for the calibration process of the stereo vision system. In the first algorithm, a Gabor filter is used to configure 4 orientations. The second algorithm is used to calibrate the individual events of cameras with four restrictions. This calibration process produces trains of corresponding event pairs. This paper included a calibration with a 2D array of blinking light-emitting diodes (LEDs). The calibration results showed a reconstruction error of only 2 mm with a standard deviation of 1 mm. The error is comparable to the size of each LED (1 mm).

#### 3.3.2. Simultaneous Localization and Mapping

Event-based cameras have also been used in the 3D simultaneous localization and mapping (SLAM) research field. SLAM requires significant computational resources, which in most cases come in the form of a GPU and as a result consumes significant power. A 3D SLAM implementation, which was composed of an embedded dynamic vision sensor (eDVS) and a frame-based depth sensor (Asus Xtion Pro Live composed of 320 × 240 pixels operating at 60 Hz) was presented in [49]. In this paper, researchers tried to solve a modified pixel correspondence problem; matching an event camera pixel with the corresponding depth sensor pixel to extract a more accurate depth result. As a limit, the corresponding pixels must be a one-pixel neighbour. The equation used for calculation of the corresponding eDVS image coordinate *u*_e_ is:

*u*_e_ = *L*^−1^(*K*_e_*TK*_d_^−1^*u*_d_), (9)

where *L* is the distortion model of eDVS, *K*_e_ is the calibration matrix for the event-based sensor, *T* is the transformation matrix, *K*_d_ is the calibration matrix for the depth, and *u*_d_ is the depth image point. For each new event, a look-up was performed in the depth map to search a one-pixel-wide neighbourhood for the smallest depth value, which was then used as the reported event depth. For this process, a dynamic Bayesian network and the condensation particle filter algorithm were utilized. The dynamic Bayesian network was used to describe system motion model *P*(*X*_k_|*X*_t−1_) and hence, no additional sensors were used. Thus, the motion model was simplified to a random diffusion process:

*P*(*X*_k_|*X*_t__−1_ = *p*_i_)≔ *N*(*p*_i_, *∑*), (10)

where *p*_i_ is the possible system state.

**Figure 5:**
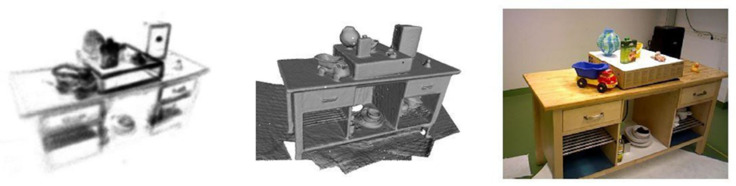
Left: The event-based 3D map M used by the algorithm in [49] for tracking. Black color indicates a high probability for event generation and thus a high score for particle evaluation. Middle: A mesh generated from the voxel-based map used by KinFu for comparison. Right: Color image for reference [49].

The map *M* was modelled as a discretized probabilistic sparse voxel grid. Each voxel in the grid indicated a probability with which a point would generate an event if the camera moved over it. Thus, voxel counted the number of events observed in certain locations yielding a simple iterative map update the rule:

(11) $$M(\lfloor \frac{1}{\lambda }p^{*}e_{k}\rceil )+=1,$$

where p* is the pose of the current best particle, *λ* is the size of voxel in world coordinates (standard is 0.01m (const.)), and *e*_k_ is a new event. The number of particles used in the particle filter has a direct influence on the runtime of the algorithm. A certain number of events were ignored to increase calculation throughput. However, this process causes instability in the algorithm. The algorithm works reliably when utilizing down to only 50% of the recorded events. Figure 5 shows an example of the event-based map generated by this algorithm.

Results showed that this implementation did not need to use a GPU. The processor runs on 17 W of power, effectively consuming only about 1 W. The algorithm needed 12 to 30 MB of random-access memory (RAM). The root mean square error (RMSE) of this algorithm extended from 3.1 cm to 13.4 cm with the highest error as a result of increasing the speed of processing to include all occurred events in a dataset. An important conclusion was reported by the authors: “An important property of dynamic vision sensors is the fact that the number of generated events only depends on the moved distance and not the velocity, but faster motions generate a higher number of events per second as the same distance traversed in smaller time”. This means that the faster camera movement in the same distance will only generate more events per second. Although, in total, the same distance will generate the same number of events. Thus, this property was used to test the method in generating more events per second by speeding up the dataset event sequence.

Fast motion can really cause motion blur in events if longer time windows are selected. The authors of [52] showed that motion blur can be solved by having a range of disparities to synchronize the position of events. A disparity image was produced in the process of fast motion, where each pixel event was estimated at a constant time with linear interpolation. However, this implementation had downsides; it required that the utilized event camera must be calibrated and rectified, so that the correspondence problem is reduced to a 1D search along the *x* dimension.

#### 3.3.3. 6-DOF Tracking from Photometric Depth Map

A 6-DOF pose tracking approach was proposed in [46] which solved the correspondence problem between an existing photometric depth map and events. In general, this paper solved two problems: pose tracking from an existing photometric depth map; and tracking the pose during very fast motion. First, the event-based pose was updated at a microsecond delay due to the inherent latency of the event-based cameras. The two poses along the sensor’s trajectory were used to correct the errors of already estimated poses. Second, the sensor’s likelihood function considered both the event generation process and the presence of noise and outliers. The sensor model (likelihood function) and motion model that were utilized were simple Bayesian filtering models, which have a correction step and a prediction step. A correction step is given as follows:

*p*(*s*_k_|*o*_1:__k_) ∝ *p*(*o*_k_|*s*_k_)*p*(*s*_k_|*o*_1:__k_), (12)

where *s*_k_ is the current state, *o*_1:k_ are all past observations, *o*_k_ is the current observation, and *p*(*o*_k_|*s*_k_) is the sensor model. The prediction step is:

*p*(*s*_k_|*o*_1:__k__−1_) = ∫ *p*(*s*_k_|*s*_k__−1_)*p*(*s*_k__−1_|*o*_1:__k__−1_)*ds*_k__−1_, (13)

where *s*_k-1_ is the previous state, and *p*(*s*_k_|*s*_k−1_) the motion model. Lastly, the posterior distribution of the system was approximated by a tractable distribution that condenses the history of events. All history observations can be condensed to some parameter *η*_k_. Subsequently, the posterior distribution can be approximated and computed using Kullback–Leibler divergence. Thus, approximate posterior distribution becomes:

*q*(*s*_k_; *η*_k_) ≈ *Cp*(*o*_k_|*s*_k_)*q*(*s*_k_; *η*_k__−1_), (14)

where *C* is some normalizing constant. Thus, the correction step is approximated to only the last observation, omitting all other past observations.

The point (pixel) in the event camera can be calculated as a point in the reference image with some error defined by a Gaussian distribution. In this way, one can calculate which pixel in the event camera corresponds to which pixel in the reference image. After this, intensity values from the reference image are taken as an approximation of the contrast. This approximation includes the current event and the last event in the same pixel. This approach is more accurate than linearizing log intensity values. Lastly, a linearization was performed around the expected state. For posterior approximation, an extended Kalman filter is used. This aided in the fusion of all the measurements and in the updating of the state vector efficiently.

In [46], the results were acquired in both indoor and outdoor experiments. The indoor experiment had a mean scene depth of 60 cm. Using eight recorded sequences, the mean root mean square (RMS) position error is 2.71% of the average scene depth. The outdoor sequences had a mean position error of the average scene depth of 3.97% (at 2.5 m) and 6.47% (at 30 m). Moreover, experiments with large scene depth variations were performed that gave 2.5–4.0% mean RMS errors in position and orientation, respectively. These results show reasonable accuracy especially given that the DVS sensor had a poor spatial resolution. However, the processing time was 32 μs for each event. Additionally, at higher motion speeds, this algorithm would have a bottleneck. Nevertheless, even for large depth variations, this algorithm performed well and should be re-tested with a higher spatial resolution DVS.

A more recent approach in visual odometry introduced novel methods of building semi-dense 3D maps and stereo rig pose tracking [50]. This work used time surfaces, that are updated with configurable time window of events, for event representation. The work contains three main modules: an event processing module, a tracking module, and a mapping module. First, the time-surface map is generated and updated with a batch of incoming events. Second, the left camera pose with respect to the local map is tracked. Finally, events, time surfaces and pose estimates are used in the final module to refresh a probabilistic semi-dense depth map. Thus, for the first time to the authors’ knowledge time surfaces were used for depth estimation, however, only semi-dense depth maps were generated.

#### 3.3.4. Spiking Neural Networks in Stereo and Monocular Depth Estimation

Artificial neural networks are not the exception when discussing depth estimation. Works utilizing SNN instantiations are most intriguing because of the asynchronous nature of operations, which is contrary to most instances of artificial intelligence use. SNNs in combination with stereo vision were used in [53], which gave depth estimation results with 96% accuracy at a maximum depth of 5 m. In another work, a belief propagation neural network, which is defined in [54], was used and a mean depth error of 61.14–92%, with a maximum depth of 5 m was reported [55]. Another proposed method included SNNs that solved the task of ascertaining DFD [28]. It should be mentioned that this method is monocular and used one of the biggest advantages of neuromorphic vision—precise timing of spikes. It has been shown both theoretically and practically that the precise timing of spikes allows neurons to perform computation with a single spike per neuron. The authors of [28] created a real-time event-based visual processing system leveraging this advantage.

The use of techniques called depth from focus (DFF) or DFD has great advantages because it can be realized in a monocular camera. Here, an event-based camera was used with a variable motorized focal lens controlled at 100 Hz. The main principle of operation was that when the lens starts working, objects will appear out of focus, then in focus and out of focus again. Hence, a given object at a given distance at some point in time will be sharp, but the rest of the time it will be blurred or out-of-focus. When the object is in focus it will produce sharp edges. Beyond the hyper-focal length, the whole scene appears out of focus, and depth information can no longer be distinguished. To compute the object’s depth information the defocus blur spot was used. To ascertain the size of the defocus blur at the focal plane following formula was used:

(15) $$s(t)\;=\;\frac{f^{2}}{N}\times \frac{\left|z\left(t\right)-d\right|}{\left(d-f\right)z\left(t\right)},$$

where *f* is the focal value of the optical system, *N* the numerical aperture, *d* the position of the object when in focus, and *z*(*t*) the variable position of the object over time (or in other words the depth). Due to the sensor and lens errors, the blur spot is further distorted by a Gaussian function.

Because the events are spread out within a Gaussian distribution the events will at some time switch from -1 (OFF) to +1 (ON). When the focus is reached the intensity change will be equal to 0. After this, a polarity change in events is followed. The detection of focus, again, is determined by time *t*_f_ which can be estimated from the average timing between consecutive ON and OFF events. After this, the depth information can be calculated by using the below equation:

(16) $$z(t_{f})\;=\;\frac{\pm \frac{df^{2}}{N}}{S\left(t_{f}\right)\left(d-f\right)\;\pm \;\frac{f^{2}}{N}}.$$

**Figure 6:**
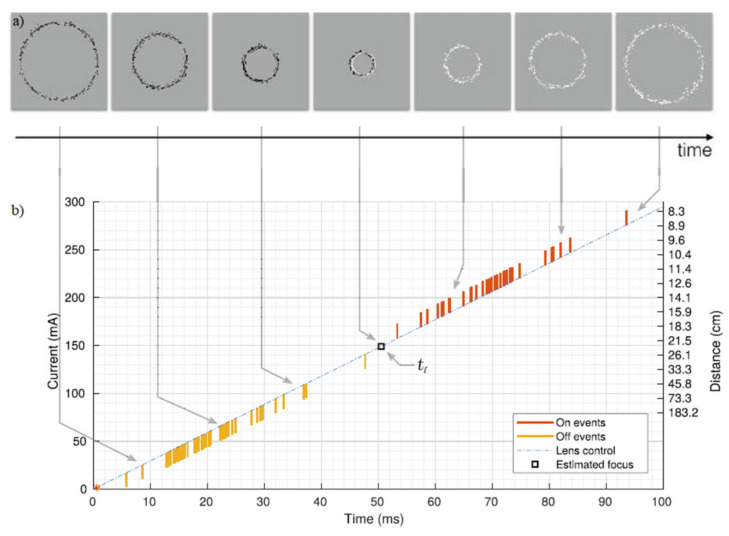
(**a)** Events corresponding to the sweeping of the focus range, in black are OFF events and in white ON events. (**b**) Representation of spikes among a single pixel, according to the driving current of the liquid lens. Here, the focus point is estimated to be 22.6 cm from the sensor [28].

The change of sign in *z* corresponds to the focal length that is the closest to the focus. Parameters *d* and *f* are controls of the liquid lens device. The average timing *t*_f_ between two consecutive events is visualized in Figure 6.

**Figure 7:**
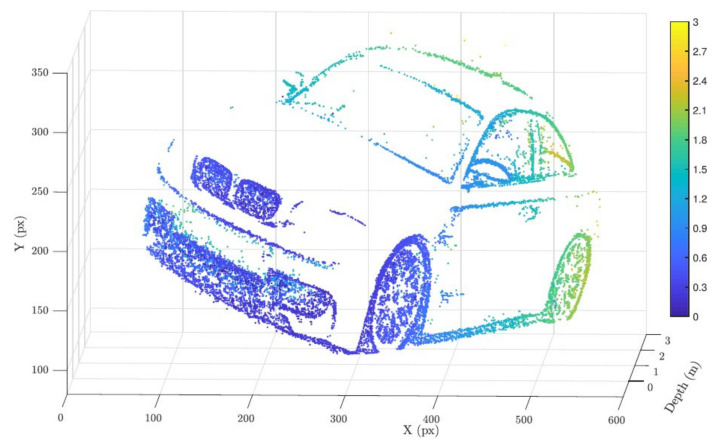
Reconstructed depth scene for the car. The depth is also color-coded for clarity [28].

The spiking neural network was composed of five neurons for each pixel. This neural network had two input neurons (one for ON events and one for OFF events), two blocker neurons that were inserted to avoid superfluous firings of the output, and finally an output neuron (OUT). The SNN was based on a Leaky Integrate-And-Fire neuron model. To estimate *t*_f_ for each pixel, the smallest time interval between two consecutive events of opposite signs was observed. First, the input ON neuron required two consecutive spikes to trigger the blocker neuron. An inhibition link to the OUT neuron ensures that the OUT neuron would not fire. After the focus, a polarity inversion occurs and the OFF neuron is fired, thus exciting the output neuron, which also subsequently fires. Again, an inhibition link ensures that subsequent OFF spikes do not trigger the output neuron. Finally, the synchronization with the liquid lens neuron (Sync neuron) was triggered by the liquid lens, indicating that the sweep was over resetting the OUT neuron to its initial state. The depth can then be extracted as the timing between the OUT and Sync spikes. Figure 7 shows a generated depth map of a sweep on the car. The depth information is represented in different colours. Some errors can be observed on the license plate of the car.

The depth results were obtained from sequences of ground-truth depth that ranged from 0.12 to 5.5 m. The network used 999,045 neurons. Neurons were implemented using the PyNN framework and simulated using the NEST neural simulator. A lens sweep of a car was performed, which generated a depth map with a mean relative error of 10.4%. Other experiments with ground truths of up to 1.5 m produced a relative error of 4% and increased up to 23% at 2 m. The authors concluded that this result was expected because the optimal system’s focal length had reached the hyperfocal distance. The presented algorithm consumed and processed 15 million events per second. The power consumption was within 200 mW (10 mW for the camera, 90 mW for the liquid lens and ~100 mW for the computation), which is quite impressive given the power consumption of other hardware instantiations.

#### 3.3.5. Monocular Dense Depth

Another source of monocular vision is presented in [56]. This approach is different from the previous method because it uses spiking neural networks to predict depth. One of the pioneering papers that presents depth prediction at every pixel, facilitating a dense depth map is [56]. This has only been done before in [57], but the approach was different, i.e., it used a stereo setup. A different approach with unsupervised event-based learning and monocular depth sensing was presented in [58]. In [58] the researchers could only extract depth information at the reconstructed edges of the image (semi-dense).

The study in [56] demonstrates how to generate dense depth maps with monocular vision, overcoming the sparsity of the event stream. The most difficult problem faced in this method is that events are generated only with intensity changes and at the edges of a moving object. Other areas remain blank resulting in the need for interpolation of regions where no data is generated. This method also exploits the temporal properties of the event stream by using its temporal consistency in combination with recurrent convolution network architecture. Thus, it will predict dense metric depths for a single monocular camera.

The main concept of the method proposed in [56] is the usage of non-overlapping windows of events that are taken at fixed intervals Δ*T*. For each window, a log depth-map prediction is processed with a recurrent convolutional neural network. The collected events in the time window Δ*T* are then encoded into *B* temporal bins; this represents a spatiotemporal voxel grid with dimensions B × H × W. The depth prediction is done with a fully convolutional neural network, based on the UNet architecture [59]. The network itself outputs a dense depth map and the metric depth can be recovered by performing the following operations:

*D*_m,k_ = *D*_max_ exp(−α(1−*D*_k_)), (17)

where *D*_max_ is the maximum expected depth, *D*_k_ is the depth prediction by model, and *α* is the parameter chosen, such that a depth value of 0 maps to a minimum observed depth. The neural network is trained in a supervised fashion. The training is performed with synthetic data and later fine-tuned using real events from the multi-vehicle stereo event camera (MVSEC) dataset [60].

**Figure 8:**
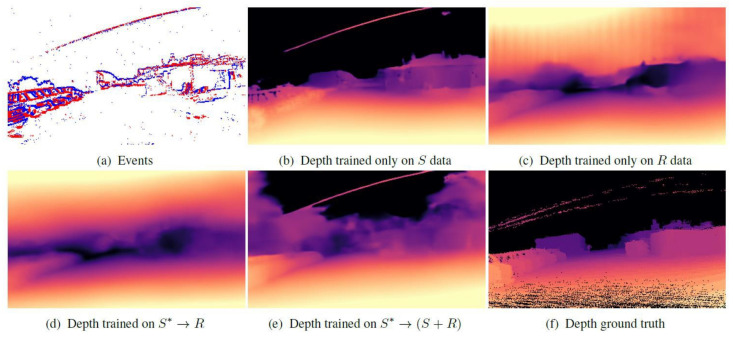
Ablation study of our method trained with different training sets: (**a**) shows the events; from (**b**) to (**e**) the predicted dense monocular depth using different training sets; (**f**) depicts the corresponding ground truth. The depth maps are shown in logarithmic scale and correspond to sample 3562 in the outdoor day-1 sequence of MVSEC [56].

The presented results consist of estimated depth errors and neural network training results. Different training sets are used. The best results are achieved when training the network with both synthetic and real data. Additionally, the monocular depth prediction accuracy grows with respect to the amount of training data available. The visualization of different types of training data is shown in Figure 8. The results from the MVSEC dataset are compared with frame-based state-of-the-art methods [61,62], and with another monocular event-based method [58]. This work outperforms all other methods at all distances—10 m, 20 m, and 30 m with an average improvement of 26.25%, 25.25% and 21.0%, respectively. Overall, this paper shows that monocular depth vision with an event-based camera can outperform other frame-based methods and is capable of estimating depth at longer distances than other stereo vision methods.

This section provided a brief overview of the depth estimation methods implemented on either a CPU or a GPU. Most developed methods utilize stereo vision systems. As expected, the provided results showed that stereo vision systems require higher computational effort and more power. Moreover, some of the methods used photometric depth maps captured by frame-based cameras, which also add additional power consumption, complexity, and equipment. On the other hand, monocular vision systems that used neural networks showed competitive results. Spiking neural networks were used to estimate depth from defocus. Moreover, a monocular, dense depth-sensing method was presented which computes depth information at each pixel. The use of this method was limited due to the lack of training datasets readily available for event-based vision.

The presented overview of these research papers showed that a variety of depth estimation methods utilizing event-based cameras exist. The division of methods based on utilized hardware showed that currently most of the research in this area is focused on standard processing units. This may be due to the desire for flexibility and scalability during the development process. Only a fraction of the currently published event-based depth-sensing methodologies utilize an FPGA or a neuromorphic processor. The utilization of a neuromorphic processor showed better overall results in computational efficiency and power consumption. Most of the papers mentioned here lack a unique dataset that would benchmark all methods equally. Most recently an MVSEC dataset [60] was proposed which simplifies the comparison of results across instantiations. Finally, both stereo and monocular, event-based camera depth-sensing implementations were introduced. However, the majority of experiments were conducted with stereo vision.

In Table 2, each referenced method is rated based on the most important parameters in depth estimation with event-based cameras. The referenced methods are rated based on range, accuracy or error, latency, power consumption and complexity. The first part of the parameters is rated based on the empirical data. The last parameter, complexity, is evaluated based on the used hardware, novelty, and the number of similar implementations. The empirical data can be viewed as better than the average if more ‘+’ symbols are added to a certain parameter. The complexity is also evaluated as less complex if more ‘+’ symbols are added to each reference. The ‘-’ symbols show that no data is presented in the reference and it cannot be evaluated compared to other methods. In conclusion, these provide the reader with a simple solution for evaluating each proposed method based on the salient performance parameters.

## 4. Results and Discussion

In this section, a summary of the results of the previously introduced methods is given. Furthermore, this summary is tabulated and described.

The most popular computational pieces of hardware selected for event-based camera depth-sensing implementations are CPUs followed by FPGAs and neuromorphic processors. This hierarchy is natural and expected given that the development process is faster, more accessible and cheaper on CPUs versus the other options. Some of the CPU-based methods presented here are directly portable to the other hardware platforms, especially the methods that require SNNs.

As expected, the presented methods demonstrate results over a variety of conditions, without a consistent benchmarking dataset, making it difficult to compare methods. However, most recently an MVSEC dataset specifically designed for benchmarking depth-sensing methods was recently published. With these limitations, a comparison of the different methods and their results is offered in Table 3. It is important to realize that the spatial resolution of the utilized camera(s) greatly impacts the depth-sensing performance. The selected hardware platforms can impact system latency, system power consumption and the portability of the method.

Table 3 presents seventeen methods that use standard CPUs, GPUs, or embedded platforms, only two methods that use FPGAs and one method that utilizes a TrueNorth processor. The table also shows that the stereo implementation is the most popular type for depth estimation. Sixteen methods used stereo vision while only four utilized monocular vision.

The latest research paper [50] performed well in a visual odometry implementation using a stereo setup with an Intel Core i7 CPU. This paper was tested on scenes with a true depth of up to 6 m and a mean error of only 0.16–0.19 m. However, the depth result latency is quite high at 78 ms. Moreover, this method estimates only sparse depth (semi-dense), which limits results only to where events occurred. On the other side, a monocular depth prediction was performed by [56] with a true depth of up to 30 m and an average depth error of 1.42–4.46 m. This method is learning-based and can predict dense depth maps (depth at all pixels).

Monocular depth vision implementations are limited. The study in [47] was the first (to the best of authors’ knowledge) to provide a 3D reconstruction of a scene based on 6-DOF camera tracking. However, only a viability study was offered without any quantifiable results with which a comparison with ground truth could be made. Moreover, this method utilized a standard CPU which would be insufficient for real-time depth monitoring of a typical scene. Nevertheless, [47] trail-blazed monocular event-based depth perception and 3D reconstruction for the next generation of efforts in this area.

Later experiments with monocular depth perception also showed promising results. The authors of [28] used a DFD method with an SNN to predict depth. The study from [28] exploited the high temporal resolution of an event camera to calculate the depth from an event stream. The method proved to be efficient and accurate in short ranges with relative error starting from 4%. However, at long ranges (>5 m) this relative error ballooned upwards towards 23%. Additionally, this method was able to estimate the depth at pixels when only events occurred. Additionally, [58] utilized an unsupervised event-based method to predict depth from optical flow. By using the edges of moving objects, [58] was able to gather depth information at the edges of an object. This was the first method to use the MVSEC dataset for benchmarking, hence it could be used for comparison with other methods assuming that the same dataset was utilized. The maximum reported distance was 30 m with an average depth error of 2.19–5.05 m. The authors of [56] created a method that used a supervised recurrent convolutional neural network. It provided a method of dense depth estimation because it predicted depth at all pixels, additionally, metric depth was also extracted. The study undertaken in [56] represents a substantial improvement for monocular depth vision, as the only other published dense depth estimation method [57] utilized a stereo rig. Although the same MVSEC dataset was used for both methods, [56] was tested outdoors and [57] was tested indoors. The results of [56] showed an average depth error of 1.42–4.46 m while [57] had an average depth error of 0.136–0.184 m.

The presented methods all achieve reasonable results. Monocular depth estimation also shows competitiveness with stereo vision. Monocular depth estimation methods [55,57] have shown good performance in outdoor scenes up to thirty meters. A stereo method [46] at this same distance had a relatively better result. However, these methods were only exercised with the available datasets and were not properly tested in practical scenarios. Moreover, the tests were not conducted under the same conditions. Supervised methods are handicapped by the requirement of more datasets to accurately predict depth in any scenario. For now, there is only one proper dataset that is suitable for supervised learning. Similarly, [56] perceived depth only on moving scenes. Pixels that observe a static scene will not generate events and thus require interpolation. This can cause difficulties predicting the depth in these blank areas. However, this limitation can be mitigated by generating events with an external source, for example, constant frequency vibrations, which can easily be post-processed. This concept is similar to animal retinas, where the vision system is stimulated by micro-eye movements [66].

In conclusion, an overview of all the methods is presented and readily tabulated above. Different depth-sensing methods utilizing different computer hardware platforms have been developed. The most popular hardware is a standard CPU and is most often used with stereo vision. Depth-sensing algorithms for event cameras struggle when the scene is static, i.e., when no events are generated, hence depth prediction cannot be achieved. On the other hand, this problem can likely be solved by using an external source, which would artificially generate events. Another major challenge for depth sensing algorithms that use supervised learning is the lack of datasets, which reduces the accuracy of perceiving depth in different scenarios.

## 5. Conclusions

The event-based camera has most recently gained traction in the commercial image sensor market as its advantages over frame-based cameras have been successfully demonstrated in some key applications. An event-based camera exhibits a significantly higher dynamic range, lower latency, and lower power consumption as compared to its frame-based brethren. However, these features have yet to be fully exploited in stereo or monocular depth vision applications. As such, event-based cameras with associated processing algorithms for depth sensing applications are still in their infancy. There are only a relatively small number of FPGA-based computational engines for processing event-based datasets. FPGA–event-based camera solutions should be better exploited for real-time processing capabilities. Neuromorphic processors that use non-Von Neumann hardware architecture have also been used in combination with event-based cameras. This amalgamation has demonstrated unprecedentedly low power consumptions and high event bandwidth performances due to the ultra-small computational latency of the aggregate data path. The most popular hardware in the event-based depth estimation field is a standard CPU which is flexible and the cheapest to use. Moreover, depth estimation methods with stereo hardware have been implemented widely. Monocular depth-sensing instantiations are significantly less prevalent, but exhibit competitive results as compared with stereo methods. The most recent research showed implementations with SNNs, unsupervised and supervised neural networks. However, these methods still exhibit limited performance due to the lack of available training datasets. More research needs to be performed in the event-based depth estimation area to fully evaluate the potential of its implementation through the development of both novel algorithms and hardware.

## Figures and Tables

**Table 2:** Referenced methods rated by the most important parameters for depth estimation. Higher number of ‘+’ symbols means better rating.

Reference	Range	Accuracy/Error	Latency	Power Consumption	Complexity
**Indoor**					
[42]	++	+	+	+	+++
[43]	+	++	+++	+	+++
[44]	-	++	-	-	+++
[51]	+	++	+	-	++
[35]	+	+	+++	-	++
[49]	-	-	-	+	+++
[48] ^1^	+	+	-	-	+++
[47] ^2^	-	-	-	-	++
[40]	+	+	++	+	+
[53]	+	++	+	+++	+
[45]	+	++	+	-	+++
[55]	+	+	++	-	++
[41]	+	++	++	++	+
[52]	+++	+++	+	-	+++
[28]	++	+	++	+++	+
[57]	+++	+++	-	-	+
[50]	++	++	+	-	++
**Outdoor**					
[46] ^3^	+++	+	-	-	++
[58]	+++	++	-	-	++
[56]	+++	+++	-	-	++

^1^ Method was mostly tested indoors with one test outdoors. Due to averaged results, this reference is designated as being tested indoors. ^2^ Method assumed to be mostly tested indoors as no experimental results included; placed as being tested indoors. ^3^ Results are designated as being taken outdoors due to having more fast motion and scene noise.

**Table 3:** Proposed methods with their results. Values are approximate due to non-standard tests performed.

Reference	Camera	Platform	Monocular/Stereo	Max Reported Depth (m)	Accuracy/Error	Latency	Power Consumption
**INDOOR**							
[42]	[63]	Embedded Blackfin BF537 DSP	Stereo	3.5	Error: 2.00–45 cm	5.0–50 ms	5 W
[43]	[15]	Standard CPU (Pentium 4 laptop)	Stereo	0.060	Error: 3.67–5.79%	0.30 ms	30% CPU load
[44]	[15]	CPU	Stereo	-	Accuracy: 95.0%	-	-
[51]	[19]	Spartan 6 FPGA	Stereo	1.0	Error: <1.50%	50 ms	-
[35]	[15]	FPGA	Stereo	3.5	Error: 0.1–0.5 m	13.7 µs	-
[49]	[64]	Single-core Intel i7 1.9 GHz CPU	Stereo	-	Error: 3.10–13.4 cm	-	17W
[48] ^1^	[15]	CPU	Stereo	5.0	Accuracy: 75–85%	-	-
[47] ^2^	[15]	CPU	Monocular	-	-	-	-
[40]	[15]	SpiNNaker	Stereo	2.8	Accuracy: 96.7–62.2%	2 ms	90 W
[53]	[21]	CPU i7 3.40 GHz	Stereo	5.0	Accuracy: 96.0%	30 ms	1.99 mW
[45]	[7]	CPU	Stereo	4.5	Error: 0.110 m	-	-
[55]	[21]	Intel i7 3.4 GHz CPU	Stereo	5.0	Accuracy: 61.1–92.0%	2 ms	-
[41]	[21]	TrueNorth	Stereo	0.12	Error: 5.00–11.6%	9 ms	0.058 mW/Pixel
[52]	[23]	NVIDIA 960M GPU	Stereo	30	Error: 0.36–0.44 m	40 ms	-
[28]	[65]	CPU	Monocular	5.5	Error: 4.00–23.0%	10 ms	200 mW
[57]	[60]	GeForce GTX TITAN X GPU	Stereo	30	Error: 0.136–0.184 m	-	-
[50]	[65]	Intel Core i7-8750H CPU	Stereo	6.0	Error: 0.16–0.19 m	78 ms	-
**Outdoor**							
[46] ^3^	[15]	Intel^®^ i7 processor at 2.60 GHz	Stereo	30	Error: 3.97–6.47%	32 μs/event	-
[58]	[60]	CPU	Monocular	30	Error: 2.19–5.05 m	-	-
[56]	[60]	CPU	Monocular	30	Error: 1.42–4.46 m	-	-

^1^ Method was mostly tested indoors with one test outdoors. Due to averaged results this reference is designated as being tested indoors. ^2^ Method assumed to be mostly tested indoors as no experimental results included; placed as being tested indoors. ^3^ Results are designated as being taken outdoors due to having more fast motion and scene noise.

## Data Availability

Not applicable.
